# Brain Inspired Dynamic System for the Quality of Service Control over the Long-Haul Nonlinear Fiber-Optic Link

**DOI:** 10.3390/s19092175

**Published:** 2019-05-10

**Authors:** Mahdi Naghshvarianjahromi, Shiva Kumar, M. Jamal Deen

**Affiliations:** Department of Electrical and Computer Engineering, McMaster University, Hamilton, ON L8S4K1, Canada; kumars@mail.ece.mcmaster.ca (S.K.); jamal@mail.ece.mcmaster.ca (M.J.D.)

**Keywords:** cognitive dynamic system, cognitive decision making, non-Gaussian and non-linear environment, situation understanding, smart systems

## Abstract

Brain-inspired intelligence using the cognitive dynamic system (CDS) concept is proposed to control the quality-of-service (QoS) over a long-haul fiber-optic link that is nonlinear and with non-Gaussian channel noise. Digital techniques such as digital-back-propagation (DBP) assume that the fiber optic link parameters, such as loss, dispersion, and nonlinear coefficients, are known at the receiver. However, the proposed CDS does not need to know about the fiber optic link physical parameters, and it can improve the bit error rate (BER) or enhance the data rate based on information extracted from the fiber optic link. The information extraction (Bayesian statistical modeling) using intelligent perception processing on the received data, or using the previously extracted models in the model library, is carried out to estimate the transmitted data in the receiver. Then, the BER is sent to the executive through the main feedback channel and the executive produces actions on the physical system/signal to ensure that the BER is continuously under the forward-error-correction (FEC) threshold. Therefore, the proposed CDS is an intelligent and adaptive system that can mitigate disturbance in the fiber optic link (especially in an optical network) using prediction in the perceptor and/or doing proper actions in the executive based on BER and the internal reward. A simplified CDS was implemented for nonlinear fiber optic systems based on orthogonal frequency division multiplexing (OFDM) to show how the proposed CDS can bring noticeable improvement in the system’s performance. As a result, enhancement of the data rate by 12.5% and the Q-factor improvement of 2.74 dB were achieved in comparison to the conventional system (i.e., the system without smart brain).

## 1. Introduction

Nowadays, the autonomic decision-making systems (ADMS) [1] for the smart interactive cyber-physical systems are attracting much attention from researchers and technology providers [1,2,3,4,5]. In this paper, a cognitive dynamic system (CDS) based on ADMS concept is presented for the fiber optic communication systems. The CDS inspired by the human brain is built on the principles of cognition, that is, perception-action cycle (PAC), memory, attention, intelligence, and language [6,7]. It has found applications in the smart home and cognitive radar. The CDS is proposed as an alternative to artificial intelligence (AI) in most AI applications [2,6,7,8,9,10]. The CDS creates internal rewards and uses it to take some actions, while AI takes actions based on the rewards from the environment. For example, the first time a child puts his hand on a hot pot, and the hand gets burned (internal reward/punishment), then the child learns that the next time he should not put his hand on a hot pot. However, in the case of AI, it would be the parent that puts the child’s hand on the hot pot for punishment (rewards from the environment). Therefore, for bringing intelligence to fiber optic communications, we choose CDS instead of AI.

CDS can be used as the brain of fiber optic transceiver. The capacity and reach of a fiber optic system are mainly limited by fiber nonlinear effects [11,12]. Some techniques have been proposed to mitigate fiber nonlinear impairments. They can be classified as (i) optical techniques, such as optical phase conjugation [13,14] and optical backpropagation [15,16,17,18]; (ii) optoelectronic techniques, such as compensation using a phase modulator [19]; and (iii) digital techniques, such as digital backpropagation (DBP) [20,21], perturbation techniques [22,23], nonlinear Fourier transform [24,25,26], and Volterra series approach [27,28,29]. The optical/optoelectronic techniques require additional photonic/optoelectronic components, while the digital techniques, such as DBP, require computational architecture with higher cost. In addition, these techniques require the fiber optic system parameters, such as the number of fiber spans, span length, dispersion, and nonlinear coefficients of each fiber span. In addition, each time a data sequence is sent, extensive signal processing is done by these techniques. The signal processing is data dependent due to the nonlinear nature of the system. Although there have been recent efforts to make the DBP adaptive [30,31], the computational cost is high. As a result, we need a technique that is adaptive, intelligent, and works without any previous knowledge about the photonics/optoelectronic components in the network. It should have a low cost and be easy to install. In addition, it should adapt itself to any fluctuations in the network such as power fluctuation or link length variations. The proposed brain-inspired dynamic system is designed to meet these requirements.

In this paper, for the first time to our knowledge, the principles of CDS are applied to a nonlinear fiber optic communication system for the bit error rate (BER) improvement and the data-rate enhancement. Some of the subsystems of the CDS, such as probabilistic shaping and Bayesian filtering, could interact coherently and systematically to improve the BER and the data-rates. The typical CDS has three main subsystems: (i) perceptor, (ii) main feedback channel, and (iii) executive. The main contribution of this paper is the design of the perceptor and executive for a long-haul nonlinear fiber optic system. The executive, the preceptor, and the feedback channel are placed in the receiver (Rx), and the executive sends action, such as new data rate by the low data rate link, to the transmitter (Tx). The basic principles of CDS are discussed in detail in [7]. The perceptor proposed here has some similarities and differences to that proposed in [7]. The typical perceptor is made up of three main subsystems: (i) Bayesian modeling, (ii) Bayesian filtering, and (iii) entropic state [7]. The perceptor presented here for fiber optic applications has some new features. The perceptor operates in two modes: (i) the prediction mode and (ii) the BER improvement mode. If the BER is less than the FEC threshold, the CDS extracts a statistical model of the fiber optic channel and saves it in the model library. Under this condition, the CDS operates in the BER improvement mode. If there is a disturbance in the fiber optic channel, the BER could increase and exceed the forward error correction (FEC) threshold. As a result, the CDS switches to the prediction mode in which the preceptor selects one of the models in the model library that is closest to the current condition. Then, the executive, in conjunction with the preceptor, attempts to bring the BER below the threshold and the CDS will switch back to the BER improvement mode. These will be discussed in more detail in Section 3, Section 4 and Section 5.

The proposed CDS can improve the BER and/or enhance the data rate based on the intelligent processing of the received data, which includes the extraction of a statistical model of the fiber optic channel or the use of the previously extracted models in the model library. Then, the perceptor sends the BER to the executive through the main feedback channel to caculate the internal reward. The executive produces actions on the fiber optic system/signal to ensure that the BER is continuously under the FEC threshold. Thus, the proposed CDS is an intelligent and adaptive system that can tackle a disturbance in the fiber optic link (especially in the optical network) using prediction in the perceptor and/or taking proper actions in the executive based on BER and the internal reward. The proposed technique based on the CDS concept has low computational cost, is a software-defined technique, and provides a significant improvement in the BER and/or the data rate. For example, in the example calculations done in Section 5, we found that the Q-factor improves by 2.74 dB and the data rate is enhanced by 12.5% using the proposed CDS system as compared to the conventional system. Additionally, unlike DBP, the proposed CDS does not require the fiber optic system parameters, such as the number of fiber spans, span length, dispersion, and nonlinear coefficients of each fiber span. Intelligent perception processing by CDS can extract a statistical model of a fiber optic channel. Moreover, the CDS can learn and recognize a disturbance in the optical network, such as a variation in data rate, fiber length, or power fluctuation. Then, the CDS using proper actions, such as data-rate tuning or probabilistic shaping of constellations, can automatically tackle network fluctuations.

The paper is organized as follows. In Section 2, the basics of CDS is reviewed. In Section 3, the concept of CDS is applied to a fiber optic system, which is nonlinear and the noise is non-Gaussian. The design of perceptor and executive for fiber optic application is discussed in detail. In Section 4, the detailed algorithm for the proposed CDS is discussed. In Section 5, the proposed CDS is implemented for a long-haul fiber optic system based on OFDM. Numerical simulations of the fiber optic system with and without CDS are carried out and the improvements resulting from the use of CDS are discussed. Additionally, the computational complexity associated with CDS is compared with other digital techniques used for fiber optic communication systems. Finally, the conclusions are presented in Section 6.

## 2. Cognitive Dynamic System (CDS)

The functional block diagram of the typical CDS is built according to the principles of cognition, that is, perception-action cycle (PAC), memory, and attention [7,8,9,10]. In the following, the role of each pillar of cognition is described in detail.

### 2.1. Perception-Action Cycle (PAC)

**Figure 1:**
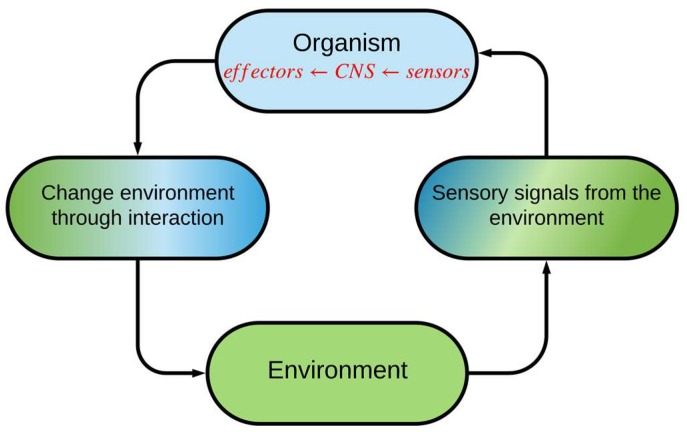
A simple diagram for perception-action cycle in the brain. (CNS: Central nervous system).

**Figure 2:**
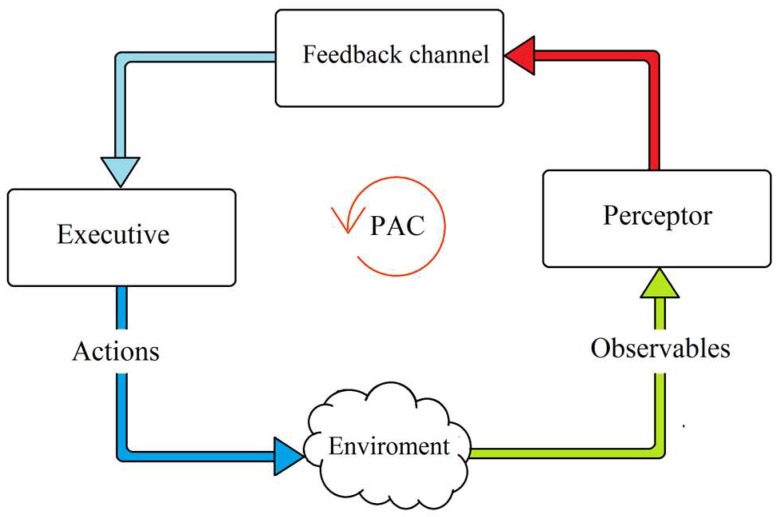
Block diagram of a basic cognitive dynamic system.

The PAC is a basic principle of cognition (Figure 1) [6]. Inspired by neuroscience and the human brain, the PAC is the cybernetic information-processing loop that helps the living organism to adapt dynamically to its environment (e.g., the environment can be the transmission medium in a fiber optic link) by aim-directed behavior or language [6]. In these activities, the CDS functions like the human brain and processes the measured information from sensors [7]. In Figure 2, the basic description of the CDS is shown. This figure gives us a better insight into how the PAC functions in the form of a global feedback loop. The PAC includes the perceptor and the executive. In addition, a feedback channel links them together, and the environment closes the feedback loop. The most important parts of PAC are the following.

1. The set of observables (which are the data obtained by measurement of the environment) are processed by the perceptor.
2. Based on the current and previous data and combined with intelligence, the perceptor predicts the states of the environment, which is passed to the executive through the feedback channel.
3. The executive produces actions on the environment to achieve a specific goal so that the set of observables in the following cycles may be different.
4. The results of each cycle of the PAC will be used for succeeding cycles.
5. When there is a specific goal, actions performed on the environment or physical system, the current PAC is guided by the derivation hypothesis from memory. As a result, the CDS will update the data in the current cycle and modify the hypothesis which will be used in the next cycle.

The actions of the executive produce a change in the environment from one state to another. This procedure continues, cycle by cycle, with further actions until the desired goal that is determined by the system policy, is achieved. The PAC may be viewed as coherent interaction and coordination between perception, prediction, action, and outcome. For example, suppose someone wants to drink a coffee (goal). If she sees a cup on the table (sense observables using the eyes), she perceives what it is and what she should do with it (perception). Even if this is the first time she is seeing this specific cup, she knows how it is different from a glass of cold water (prediction). This is because the extracted models for cups and glasses are stored in the brain and it can predict what is currently seen is a coffee cup or a glass of water based on previous experiences (extracted models of cups and glasses). The brain can also predict the hand movements before doing an action (prediction of outcome of actions), and she automatically picks up a coffee cup with her hand (action). Her eyes sense the cup and her brain measures the distance to the cup from her hand (perception which is the outcome of the action, “picking up the cup”). This process continues until the cup is close to her mouth (PAC).

### 2.2. Memory

**Figure 3:**
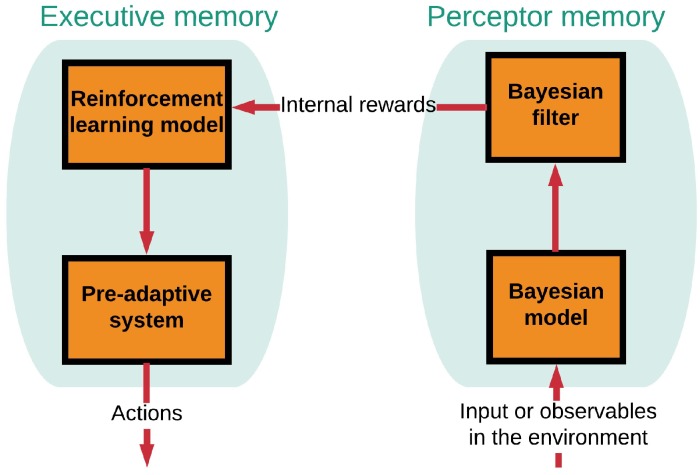
The functional brain-like block of the executive and perceptual memory in the cognitive dynamic system (CDS).

**Figure 4:**
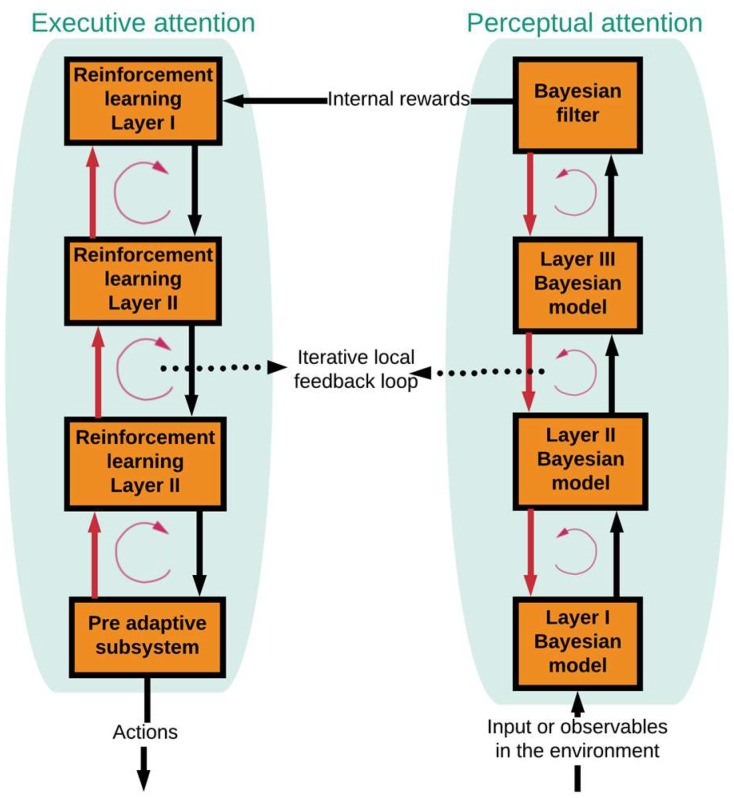
Attention (i.e., focusing) on the CDS.

Figure 3 shows the functional block diagram of a brain-like memory in the CDS [8]. The memory is required in the preceptor (known as perceptual memory) as well as in the executive (known as executive memory). While the perceptual memory enables the perceptor to recognize the distinctive features of the observables and categorize the learned features accordingly in some statistical sense, the executive memory keeps track of the chosen actions in the past and their effectiveness (Figure 3) [8]. The function of the memories is to learn from the environment, store the acquired knowledge, continually update the stored knowledge in the presence of environmental fluctuations, and predict the consequences of actions taken and/or selections made by the CDS [7]. As shown in Figure 4, first, a statistical model (Bayesian model) of the environment is established, which is followed by the Bayesian filter for estimating the environment. The Bayesian model is responsible for feature extraction. Internal rewards (positive or negative) are sent from the preceptor to the executive. The reinforcement-learning model in the executive exploits the internal reward attributed to imperfection in the preceptor. As a result, the best possible actions are chosen based on internal rewards (positive or negative) [7]. 

## 3. Applications of the CDS for the Fiber Optic Link

**Figure 5:**
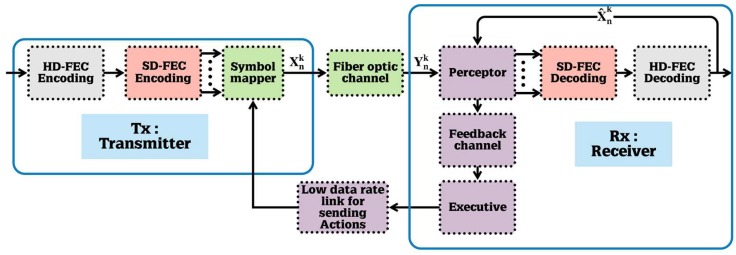
The basic design for quality of service (QoS) control and bit error rate (BER) improvement by CDS for the fiber-optic link. (HD-FEC = hard decision forward error correction, SD-FEC = soft decision forward error correction).

**Figure 6:**
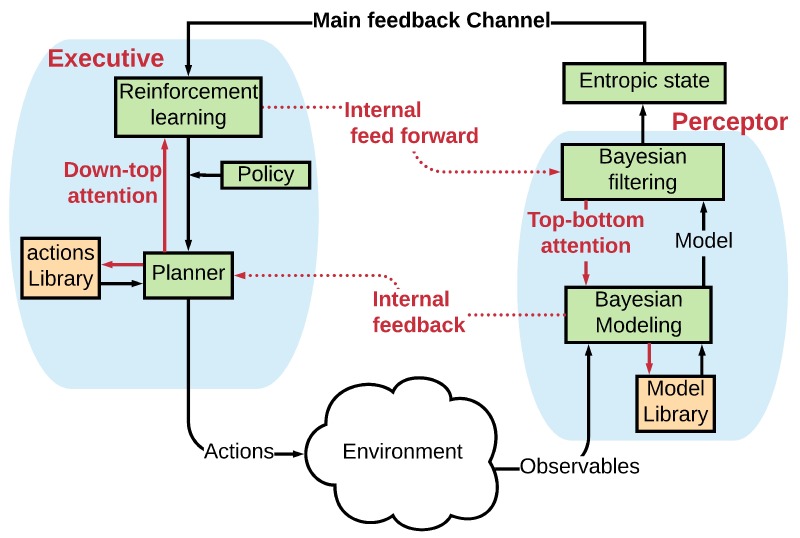
Block diagram of a typical CDS.

**Figure 7:**
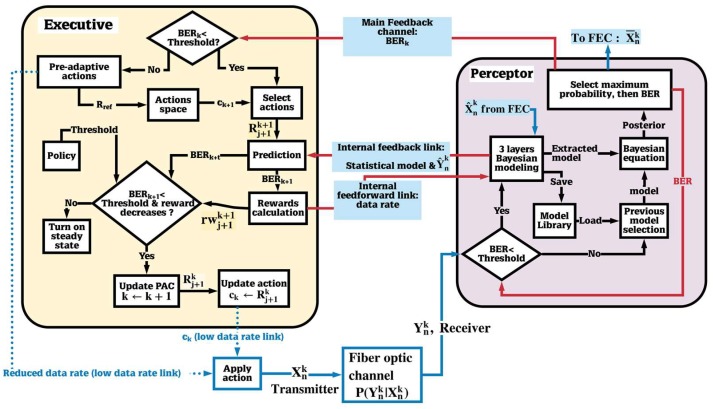
The proposed CDS for the long-haul fiber optic link.

Here, applications of the CDS to the fiber optic link is presented. Figure 5 shows the block diagram of a fiber optic system with CDS. The executive, the perceptor, and the feedback channel are placed in the receiver (Rx), and the executive sends actions such as new launch power to the fiber optic link by low data rate link to the transmitter (Tx). In Figure 6, $Y_{n}^{}$ and $X_{n}^{}$ are received and transmitted symbols, respectively, and ${\hat{X}}_{n\;}^{}$ is the received symbols after forward error correction (FEC). Moreover, Figure 6 and Figure 7 shows the block diagram of the proposed CDS in this paper. In addition, we describe the blocks in Figure 7 in Section 3.1, Section 3.2 and Section 3.3 for the perceptor, feedback channels, and executive respectively.

### 3.1. Perceptor

Typically, the perceptor is made up of three main subsystems: (i) Bayesian modeling, (ii) Bayesian filtering, and (iii) entropic state (see Figure 6). However, the perceptor proposed here (see Figure 7) for fiber optic application has more features than those discussed in [7]. The proposed perceptor operates in two modes: (i) prediction mode and (ii) BER improvement mode. If the BER is less than the FEC threshold, the CDS extracts a statistical model of the fiber optic channel and saves it in the model library. Under this condition, the CDS operates in the BER improvement mode. If there is a disturbance in the fiber optic channel, the BER could increase and exceed the FEC threshold. Now, the CDS switches to the prediction mode in which the perceptor selects one of the previous models that is closest to the current condition. The executive, in conjunction with the perceptor, attempts to bring the BER below the threshold and then the CDS will switch back to the BER improvement mode. 

As shown in Figure 6 and Figure 7, the input of the perceptor block is the received symbol $\;Y_{n}^{k}$. The index *k* denotes the cycle number of PAC. As discussed later, the main sub-blocks of the proposed perceptor are: (i) three layered Bayesian generative model, (ii) previous model selection, and (iii) Bayesian filter.

#### 3.1.1. Three-Layered Bayesian Generative Model

**Figure 8:**
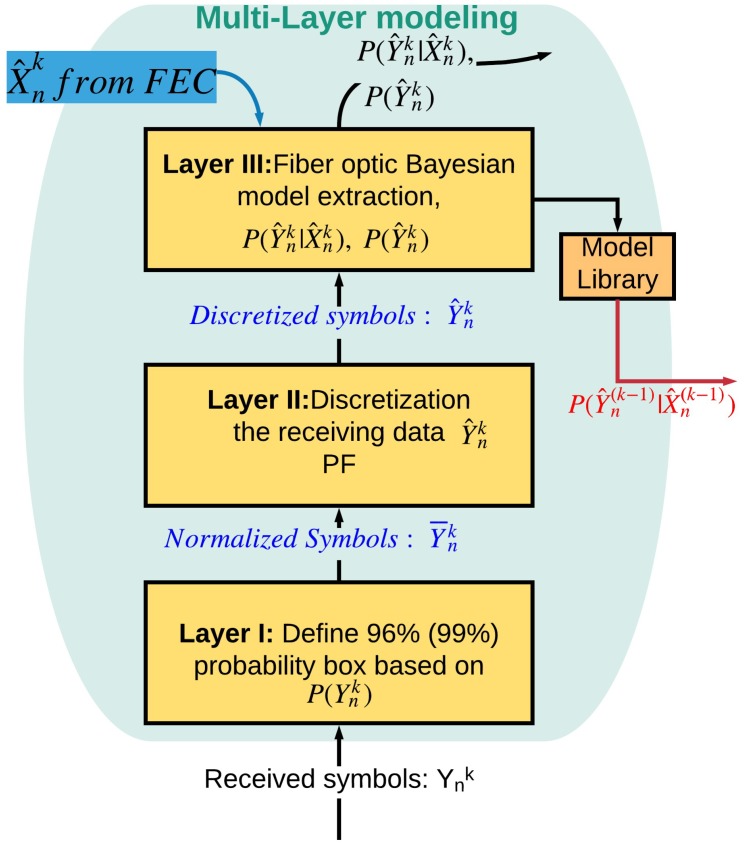
Three layered Bayesian modeling inspired by the brain for modeling fiber optic link.

Figure 8 shows the three-layered Bayesian modeling for a long-haul fiber optic system inspired by the human brain. Suppose that, initially, there exists no statistical model of the system in a model library. Then, the three-layered Bayesian modeling block extracts the statistical model of the system. We illustrate the modeling process by performing the numerical simulation of the OFDM system. The simulation parameters are presented in Table 1. 

The signal propagation in an optical fiber is described by the nonlinear Schrodinger equation (NLSE), which can be solved using the standard split-step Fourier scheme [32,33]. The output of the fiber optic link passes through a coherent receiver and then the Fast-Fourier transform (FFT) is applied to demultiplex the subcarriers of the OFDM data (See Section 5 for more details). The channel estimation and compensation of linear impairments is done using the one tap linear equalizer that uses 16 training frames to mitigate linear distortion [33]. The output of the linear equalizer is passed to the preceptor. $X_{n}^{k}$ represents the transmitted QAM-16 symbols which takes any one of the values $X_{t}$ from the following set {±3±3j, ±1±1j, ±1±3j, ±3±1j}, with equal probability at the *k*th cycle and at discrete time *n*, and *t* is the index denoting one of the 16 possible symbols. $Y_{n}^{k}$ represents the corresponding received symbols after linear equalization. The three-layered Bayesian modeling consists of three layers, as explained below.

##### Layer I

**Figure 9:**
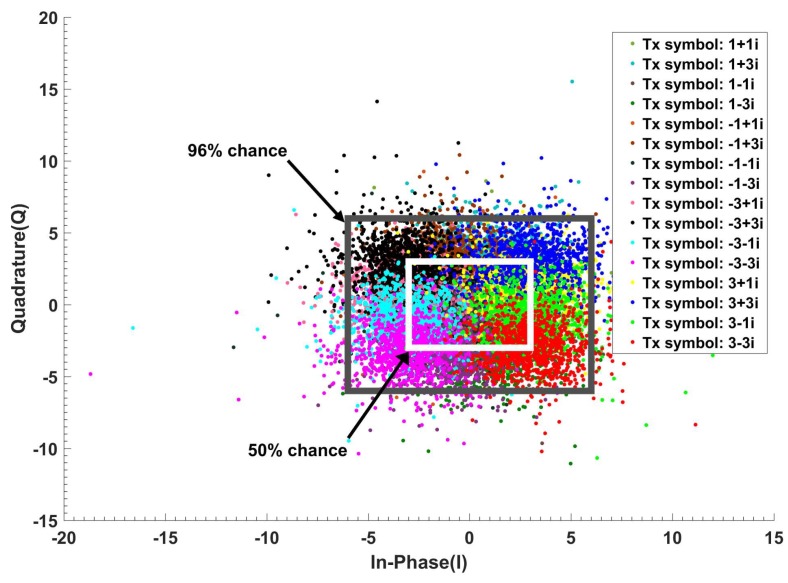
Received symbols ($Y_{n}^{k}$) after linear equalization from the simulation results.

Figure 9 shows the in-phase (I) and quadrature (Q) of the received data $Y_{n}^{k}$. We define 96% probability box (or 99% probability box depending on the application), which means that 96% of the received data is inside this box when QAM-16 data is sent. In addition, the system maps and normalizes all receiving symbols outside of this box to its borders. For example, if the received symbols $Y_{n}^{k}$ is 8 + 9*j*, then it is mapped to 6 + 6*j* when the 96% box is used. The normalized data ${\bar{Y}}_{n}^{k}$ (i.e., 6 + 6*j* in this example) is sent to Layer II (Figure 8) for further processing. The BER is used to adaptively select the borders of the probabilistic box. For lower computational cost and faster modeling, a probability box with smaller area is better. For example, the computational cost associated with the 90% probabilistic box is a lower than the 99% probabilistic box. However, this leads to inaccurate results and higher BER. Therefore, the BER is used as a feedback to choose the optimum probability box for which the computational cost is lower with acceptable high accuracy (in this example, it is 96% probabilistic box, see Figure 9). 

##### Layer II

**Figure 10:**
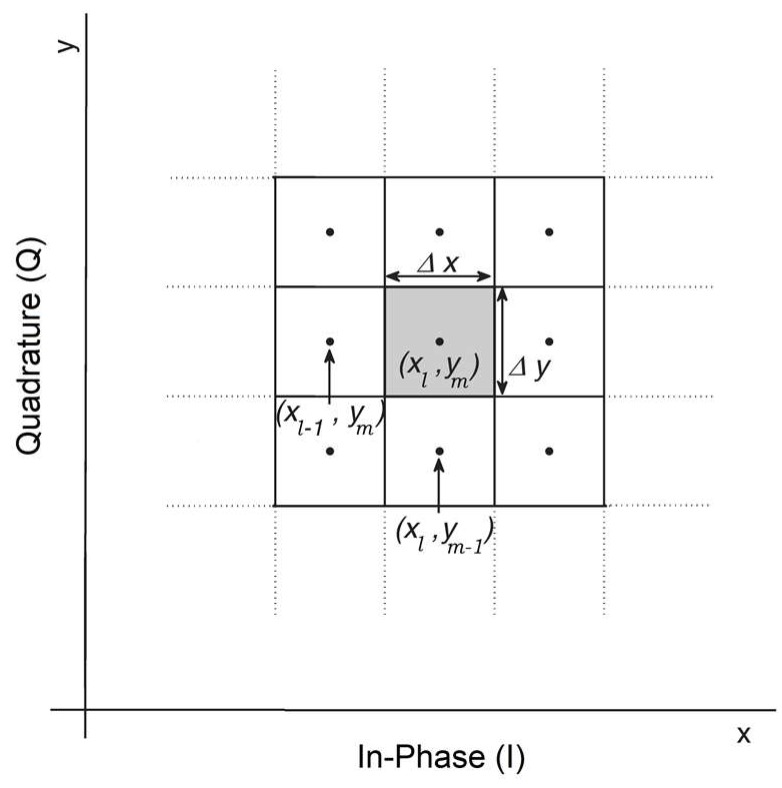
Discretization of received symbols.

**Figure 11:**
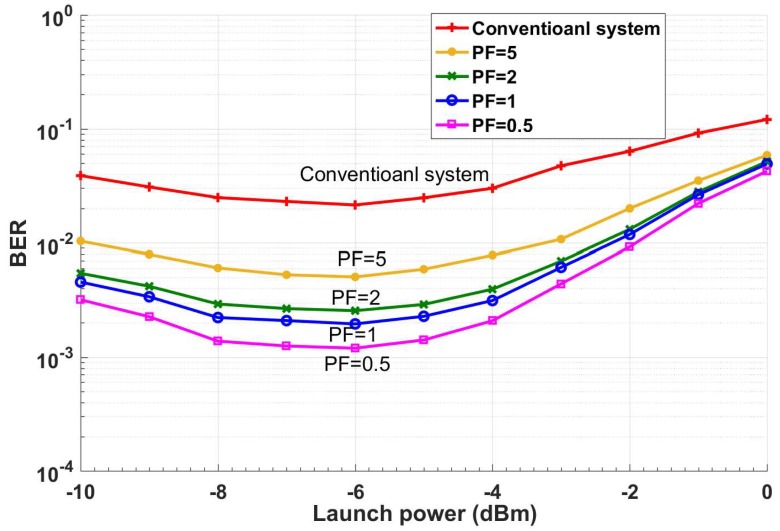
The simulation of BER improvement for various precision factor (data rate = 52 Gb/s and transmission distance, *L* = 1600 km).

In Layer II, the received symbols are discretized with discretization steps Δx and Δy. For simplicity, we have assumed Δx=Δy and we define precision factor (PF) = 10Δx=10Δy. The normalized received symbols, ${\bar{Y}}_{n}^{k}$ that are inside the shaded box (see Figure 10) are mapped to the center of the square as $\left(x_{l,},\;y_{m}\right)$, where *l* is the index for in-phase and *m* is the index for quadrature. For example, if $x_{l}=1$, $y_{m}=2$ and ${\bar{Y}}_{n}^{k}=1.1+2.05j$, it is mapped to ${\hat{Y}}_{n}^{k}=$ 1 + 2*j*. The discretized symbols, ${\hat{Y}}_{n}^{k}$ are sent to Layer III for probability estimation. This discretization is inspired by the brain. Our brain monitors the temperature, light, and other environmental conditions and maps this low-level representation to our initial perception, which is a high-level representation such as day or night, cold or warm [34]. The low-level representation requires a huge amount of data, which is mapped to an important set of data (initial perception) that takes significantly less memory. This discretization can be more complicated if we use non-uniform discretization. Figure 11 shows the comparison between the CDS system with PF = 0.5, 1, 2, 5 and the conventional system for 52 Gb/s data rate (details on conventional system and BER calculation are provided in Section 5.1). Thus, a lower PF means a larger BER improvement and higher model accuracy. However, it leads to higher computational cost. The Layer II also receives the BER as top-bottom attention and it can choose the PF based on the BER. Layer II will send the discretized received symbols as ${\hat{Y}}_{n}^{k}$ to Layer III. 

##### Layer III

**Figure 12:**
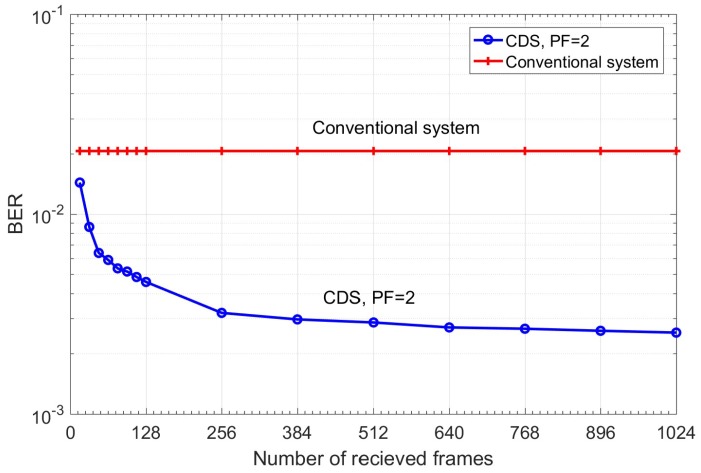
Model accuracy improvement versus a number of the received frames from the simulation results. Data rate = 52 Gb/s, *P_tx_* = −7 dBm, and *L* = 1600 km.

In this layer, the system estimates the probability of receiving $Y_{n}^{k}$ for the given transmitted symbol $X_{n}^{k}$, i.e., $P(Y_{n}^{k}\;|X_{n}^{k})$ by approximating it as the probability of ${\hat{Y}}_{n}^{k}$ for given ${\hat{X}}_{n}^{k}$ (i.e., $P({\hat{Y}}_{n}^{k}\;|{\hat{X}}_{n}^{k}))$ using the Monte-Carlo method. The transmitted symbols $X_{n}^{k}$ could be known to the receiver by sending training symbol sequences during the statistical model extraction process. However, this would interrupt the service. Instead, we approximate $X_{n}^{k}$ by ${\hat{X}}_{n}^{k}$ which is the estimate of $X_{n}^{k}$ after the FEC. We assume that the BER under FEC threshold is10^−2^ [35]. The BER after FEC for such a threshold is between 10^−15^ and 10^−12^ [35]. We need a certain number of data frames (each frame includes 256 subcarriers) for accurate estimation of $P({\hat{Y}}_{n}^{k}\;|{\hat{X}}_{n}^{k})$. Figure 12 shows the BER as a function of the number of received OFDM frames used in the calculation of $P({\hat{Y}}_{n}^{k}\;|{\hat{X}}_{n}^{k})$. As shown, the BER converges to 2.7 × 10^−3^ after 640 OFDM frames for the CDS system with PF = 2. Therefore, it can automatically find the sufficient number of OFDM frames. For example, suppose that the number of transmitted symbols, $X_{n}^{k}=1-3j$ in a subcarrier is 100,000. Due to channel noise and distortion in the fiber optic link, they could be mapped to any point in Figure 10. Next, suppose that the number of received symbols (after normalization and discretization in layers I and II) with $Y_{n}^{k}=2.2-1.5j$ is 10,000 when the transmitted symbol is $X_{n}^{k}=1-3j$. Layer III counts these 10,000 received symbols are counted and calculates $P\left({\overset{⌢}{Y}}_{n}^{k}=2.2+1.5j|{\overset{⌢}{X}}_{n}^{k}=1-3j\right)=0.1$. In other words, in Layer III, the number of hits in each of the cells of Figure 10 is calculated and the calculated probability $P({\hat{Y}}_{n}^{k}\;|{\hat{X}}_{n}^{k})$ is stored in a tensor whose dimension is shown in the second column of Table 2. The probability calculation is done for each type of the transmitted symbol. For example, QAM-16 has 16 constellation points or 16 different types of transmitted symbols. The tensor shown in Table 2 has three dimensions. In Table 2, first, second, and third tensor dimensions correspond to discretized cells for In-phase (I) axis, quadrature (Q) axis (after 2D discretization in Layer II, see Figure 9 and Figure 10), and number of constellation points of QAM, respectively. For example, if we consider PF = 5 in the first row of Table 2, then 33 × 29 × 16 corresponds to 33 columns (I-axis), 29 rows (Q-axis), and QAM-16, respectively. In addition, PF = 5 means the discretization step is 0.5 (see Section 3.1.2).

From Table 2, we see that the required memory for PF = 0.5 is 16 times more than that for PF = 2. As can be seen from Figure 11, the BER improves as PF decreases, but a lower PF implies a larger memory (see Table 2). Hence, there is a trade-off between the BER improvement and memory requirements. The extracted model in this layer can be called the statistical model for a fixed fiber network with specific signal and system parameters. This model is saved in the model library for future use. This model is not data dependent, although it has labels for various data rates, fiber lengths, launch power, or other system parameters such as nonlinear coefficient or span length. Unlike the DBP method, the proposed CDS does not need to perform extensive data dependent signal processing if the fiber optic channel does not change appreciably. The extracted statistical model will be used for prediction if there is a disturbance in a fiber optic network, such as a power fluctuation or a change in transmission length due to re-routing. 

For model extraction, we do not require training frames. Instead, we use the FEC output as an approximation to the transmitted symbol, and the executive controls the data rate to ensure that the input BER of FEC is always less than the FEC threshold and the data can be transmitted continuously without interruption. For example, when users watch live video on YouTube, the video quality automatically varies (which corresponds to variable data rate) for good quality-of-service (QoS) and minimal interruptions.

#### 3.1.2. Previous Model Selection

If the BER exceeds the FEC threshold, then $P({\hat{Y}}_{n}^{k}\;|{\hat{X}}_{n}^{k})$ cannot be extracted by the three-layer modeling in BER improvement mode. In this case, the system, like a human brain, needs prediction. Thus, it is necessary to use previous experiences for predicting the future. As a result, this subsystem (see Figure 7) will choose the closest model to the current system state (state number *k*). Suppose we have selected the model $P({\hat{Y}}_{n}^{k-1}\;|{\hat{X}}_{n}^{k-1})$. The model (*k* − 1) could correspond to one of the previous data rates, input launch power, previous transmission distance, etc. For example, if the transmission distance is changed from 1600 to 1800 km due to re-routing in fiber optic network, but the data rate, and launch power are fixed at 52 Gb/s, and –6 dBm respectively, then this subsystem will select the closest model to the current system state for prediction (closest in reach, launch power, and data rate). For more accurate prediction, the perturbations to the system should be small, in other words, the changes in transmission reach $\Delta L\;\left(L_{k}-L_{k-1}\right)$ and/or the change in data rate $\Delta R\;\left(R_{k}-R_{k-1}\right)$ should be small enough (e.g., ΔR = 2 Gb/s when the data rate is 50 Gb/s.) The CDS system will find the closest reach to the current reach and/or closest data rate from the model library for prediction. We considered *k* to be scalar for simplicity. In general, *k* could be a vector, [*l*, *m*, *n*, *p*, …]. For example, *l*, *m*, *n*, *p* could correspond to data rate, launch power, fiber nonlinear coefficient, and transmission reach, respectively.

#### 3.1.3. Bayesian Equation

The Bayesian equation is another subsystem that is shown in Figure 7. If the BER is under the FEC threshold, $P(X_{n}^{k}\;|{\hat{Y}}_{n}^{k})$ model extracted in Layer III will be used and the CDS operates in the BER improvement mode. In this mode, the estimation is as follows,

(1) $$P(X_{n}^{k}\;|Y_{n}^{k})≅\frac{P({\hat{Y}}_{n}^{k}\;|{\hat{X}}_{n}^{k})P(X_{n}^{k})}{P({\hat{Y}}_{n}^{k})}$$

Here, the index *k* is PAC number in CDS. If the BER exceeds the FEC threshold, the CDS will use the model $P({\hat{Y}}_{n}^{k-1}\;|{\hat{X}}_{n}^{k-1})$ stored in the model library to approximate $P^{k}({\hat{Y}}_{n}^{k}\;|{\hat{X}}_{n}^{k})$ as predictive Bayesian model:

(2) $$P(X_{n}^{k}\;|Y_{n}^{k})≅\frac{P({\hat{Y}}_{n}^{k-1}\;|{\hat{X}}_{n}^{k-1})P(X_{n}^{k})}{P({\hat{Y}}_{n}^{k})}$$

The model $P({\hat{Y}}_{n}^{k-1}\;|{\hat{X}}_{n}^{k-1})$ is the closest model to the current system state. Hence, the CDS can guarantee maximum possible data rates using the prediction based on previous models and improve the BER. In statistical processing using the Bayesian approach, it is well known that the evidence $P({\hat{Y}}_{n}^{k})$ can be considered as the scaling factor. Therefore, the evidence can be neglected [7,8,9,10]. 

We accomplish the task of model extraction by cycling through the prediction mode and BER improvement mode, as will be explained in Section 5.

#### 3.1.4. Selecting Maximum Probability and BER Calculation

The block selecting maximum probability in perceptor (see Figure 7) is equivalent the entropic-state part of the preceptor in typical CDS (see Figure 5). The CDS can save the posterior probability $P(X_{n}^{k}\;|Y_{n}^{k})$ in the perceptor library after one-time calculation and it is not necessary to calculate Equation (1) and Equation (2) for each symbol. The symbol that has the maximum probability to be transmitted is selected using

(3) $${\bar{X}}_{n}^{k}=\underset{t=1,2,\ldots ,M}{\arg\max}\{P(X_{n}^{k}=X_{t}|{\hat{Y}}_{n}^{k})\}$$

where, *t* = 1, 2, …, *M*, and *M* is the constellation size. For example, for QAM-16, *M* is 16, and $P(X_{n}^{k}=X_{t}|{\hat{Y}}_{n}^{k})$ represents the probability that the transmitted symbol $X_{n}^{k}$ is $X_{t}$ which is one of the values from the symbol alphabet {±3±3j, ±1±1j, ±1±3j, ±3±1j} for the given ${\hat{Y}}_{n}^{k}$. The symbol ${\bar{X}}_{n}^{k}$ that has the highest chance to be transmitted for the given ${\hat{Y}}_{n}^{k}$ is selected. Comparing ${\bar{X}}_{n}^{k}$ and ${\hat{X}}_{n}^{k}$, the BER is computed by error counting and the BER is sent to executive. The extrinsic information transfer (EXIT) chart is used to find the BER before FEC [35]. As top-bottom attention, this sub-system also sends the BER to the other subsystems in the perceptor. If the BER exceeds the FEC threshold, the FEC output will not converge and the post-FEC BER will be high and, hence, the system would infer that the pre-FEC BER has exceeded the FEC threshold. Therefore, the executive will take some actions such as decreasing the data rate. In addition, the local cycles in the preceptor will examine a different type of model for prediction and try to decrease the BER as much as possible.

### 3.2. Main Feedback, Internal Feedback, and Feedforward Channels

As shown in Figure 7, the main feedback and the internal feedback channels were used to exchange the information between perceptor and executive. The perceptor sends the BER for calculating the internal reward to the executive through the main feedback channel. In addition, the perceptor sends the probabilistic model ($P({\hat{Y}}_{n}^{k}\;|{\hat{X}}_{n}^{k}))$, evidence ($P({\hat{Y}}_{n}^{k})$) and current perceptor mode to the executive through the internal feedback channel. The executive sends information to the perceptor through feedforward channel such as OH (overhead percentage of forward error correction (FEC)) and new constellation geometry (if geometric shaping is used), fiber launch power *P_tx_*, data rate, and $P(X_{n}^{k})$ (if probabilistic shaping is used).

### 3.3. The Executive

The executive is placed in the receiver (see Figure 6 and Figure 7), but it can send the action to the transmitter, such as new data rate by low data rate link. Based on the received BER sent through the main feedback channel as raw reward, together with the probabilistic model ($P({\hat{Y}}_{n}^{k}\;|{\hat{X}}_{n}^{k}))$ and evidence ($P({\hat{Y}}_{n}^{k})$) sent through the internal feedback, the executive may decide to increase or decrease the data rate. It could adjust the launch power, modulation format, soft decision (SD) or hard decision (HD), overhead (OH) percentage, code words value, and probabilistic and geometric shaping with a desired FEC threshold for acceptable redundancy. The executive can do two types of actions based on “actions space” block in Figure 7: (i) basic actions and (ii) advanced actions, which are discussed below.

#### 3.3.1. Basic Actions

These actions include adjusting launch power and data rates in fixed or variable discrete steps. The executive will decide what actions to take based on the received BER sent through the main feedback channel. When there is a disturbance in a fiber optics communication system such as power fluctuation or reach change, and if there is no proper model in the perceptor library that is closest to the perturbed system, then the executive will become aware of it through the BER sent through internal feedback channel (in this case, the BER will be high). The executive will take basic actions such as lowering the data rate so that the BER is below the FEC threshold. When the desired goals are met, the executive would save the corresponding actions in the actions library and will load them for the similar situations in future.

#### 3.3.2. Advanced Actions for BER Improvement

One of the important challenges in fiber optic communications is to evaluate the Shannon capacity in the presence of fiber nonlinear distortion [36,37,38]. Most current optical systems use the input signal distribution whose alphabets occur with equal probability. The performance of such systems can be improved by using more advanced input signal distributions. For example, improvement can be achieved by using geometric modulation shaping or probabilistic modulation shaping [36]. The BER being less than but close to the forward-error-correction (FEC) threshold is not desirable in practical applications because of the additional margin required to account for fluctuations in the fiber optic link [38]. Therefore, the system should do some actions to improve the BER in a fiber optic link. The advanced actions in actions space are the probabilistic modulation shaping and geometric modulation shaping that the executive uses to improve the performance of a nonlinear system. Probabilistic modulation shaping refers to uniform constellation points with the non-uniform probability distribution of symbols whereas geometric shaping refers to non-uniform constellation points with the uniform probability distribution of symbols [38]. Probabilistic shaping can be performed using modified FEC codes [39,40]. However, these actions are not done blindly by CDS, and it can be done based on the desired goal to maximize $P(X_{n}^{k}\;|Y_{n}^{k})$ based on taking actions to change $P({\hat{Y}}_{n}^{k})$ and $P({\hat{Y}}_{n}^{k}|{\hat{X}}_{n}^{k}),$ by mapping to different constellation geometry or changing $P(X_{n}^{k})$ (probabilistic shaping) using Equation (2).

#### 3.3.3. Policy

The policy determines the goals that the CDS should achieve. The first goal is that the BER should be under the FEC threshold. The second one is for an acceptable computational cost. The storage or computational cost depends on PF and ΔBER (Figure 12). We define ΔBER as the difference in BER between current received frames and previous received frames with a step of 128 frames. For example, we can set our policy that the desired ΔBER should be less than 5 × 10^−5^. From Figure 12, we find that if the current received frame is 768 and the previous received frame is 640, then ΔBER= 4 × 10^−5^, which is less than the desired ΔBER. Therefore, the CDS stops the model extraction process after receiving 768 frames and saves the model and the posterior probability in the CDS model library.

#### 3.3.4. The CDS with a Simple Executive

In this section, we present the CDS with a simplified executive that does only basic actions. Figure 7 shows the block diagram of the proposed executive for the long-haul fiber optic link. In Figure 7, the executive receives the *BER_k_* through the main feedback channel at the *k*th cycle. We could define the internal reward as a function of the *BER_k_* only. However, in general, as the data rate increases, the BER increases too, and higher data rates are desirable while the BER is relatively low, (i.e., under the FEC threshold). Hence, a better definition of internal reward should be a function of both BER and the current data rate. Therefore, we define internal reward as follows:

(4) $$rw_{j}^{k}=\frac{BER_{k}}{{\left(R_{j}^{k}-R_{ref}\right)}^{4}}$$

(5) $$R_{j}^{k}=R_{ref}(Gb/s)\;+j\times d(Gb/s)$$

where the *BER_k_* is the BER at *k*th PAC, *d* is the discretization step for changing the data rate which is set equal to 2 Gb/s in this paper, and *j* is equal to *k* before a disturbance to the fiber optic system and it is reset to 1 after the disturbance and pre-adaptive actions. The pre-adaptive actions refer to those actions which reduce data rates until the BER is under the FEC threshold. $R_{ref}$ is a convenient reference data rate that is set equal to $R_{1}^{k}-d$. The executive updates the $R_{ref}$ after any disturbance using pre-adaptive actions. The internal reward $rw_{j}^{k}$ and data rate $R_{j}^{k}$ are further explained in Section 5.2 with an example.

The actions space for this specific case study can be defined for the fiber optic link as follows:

(6) $$C=\{c_{k}|data\;rate\;R_{j}^{k}>R_{ref}^{}\}$$

where ***C*** is set of all possible actions in the actions space, and $c_{k}$ is the action at *k*th PAC that belongs to the action space ***C*** such that the data rate $R_{j}^{k}$ exceeds the reference data rate $R_{ref}$. 

Suppose that the current action is $c_{k}$.The executive can predict the outcome of the prospective action $c_{k+1}$, before applying it on the fiber optic channel using the following equations:

(7) $$P(X_{n}^{\left(k+1\right)}|{\hat{Y}}_{n}^{k})=\frac{P({\hat{Y}}_{n}^{k}|X_{n}^{\left(k+1\right)})P(X_{n}^{\left(k+1\right)})}{P({\hat{Y}}_{n}^{k})},$$

(8) $${\bar{X}}_{n}^{\left(k+1\right)}=\underset{t=1,2,\ldots ,M}{\arg\max}\{P(X_{n}^{\left(k+1\right)}=X_{t}|{\hat{Y}}_{n}^{k})\},$$

where $X_{n}^{\left(k+1\right)}$ represents the input symbols virtually generated at the executive at (*k*+1)th PAC at the data rate $R_{j+1}^{k+1}$. The BER is computed by comparing ${\bar{X}}_{n}^{\left(k+1\right)}$ and $X_{n}^{\left(k+1\right)}$. Note that there are two types of predictions, one at the preceptor and the other at the executive. When the BER exceeds the FEC threshold, the preceptor switches to prediction mode and selects the previous model closest to the current situation. When the BER is under the FEC threshold, the executive enhances the data rate to $R_{j+1}^{k+1}$ and predicts the BER using Equation (7) and Equation (8) (see Figure 7).

The subsystems of the executive shown in Figure 7 are explained as follows. If the BER exceeds the FEC threshold, the executive runs pre-adaptive actions such as data rate reduction and sending the updated reference data rate $R_{ref}$ to the actions space. If the BER is less than FEC threshold, the executive selects an action from the actions space. Here, the action is the data rate enhancement. The block “prediction” in the executive predicts the *BER_k+1_* based on the statistical model received from the preceptor through the internal feedback link (see Equation (7) and Equation (8)). The block “rewards calculation” computes the internal reward using the predicted BER and a prospective data rate of $R_{j+1}^{k+1}$ and Equation (4). If the predicted BER is below the FEC threshold and a lower value of the internal reward is achieved, then the executive interprets that the system is performing well with $R_{j+1}^{k+1}$, and the data rate can be enhanced further. The new (prospective) data rate is communicated to the transmitter using a low data rate link. Otherwise, the executive turns on the steady state mode, and assumes $R_{j}^{k}$ is the best possible data rate that can be achieved for the current fiber optic link.

## 4. The Algorithm of CDS with a Simplified Executive

In this section, we describe the proposed algorithm for the simplified CDS applied on a fiber optic link. Algorithm 1 shows the outline of the complete one PAC procedure of a simplified CDS. In addition, Table 3 lists all the notations used in this paper. 

The algorithm is described briefly as follows:

1. Prediction of rewards using available models or reduction of the data rate until the BER is less than the FEC-threshold.
2. Extract the exact model of the system and minimize the BER.
3. Use this model to predict the BER and internal rewards before increasing the data rate.
4. If prediction shows lower internal reward and the BER is less than FEC-threshold, then the CDS increases the data rate.

**Algorithm 1:** Environmental actions through the global feedback **Input:** the observables $Y_{n}^{k}$**Output:**$c_{k+1}$ as the final action in environmental actions mode  **Initialization:**
  *Pre-adaptive actions:*  *Reduce data rate till BER<FEC-threshold*  Make steady state mode off, prediction mode on  *Probability box% = 96, threshold = 0.01*, PF=2, *j = 1*  
$c_{k^{\prime }+1}\leftarrow first\;action\;from\;C$
  Apply $c_{k^{\prime }+1}$ to the environment (fiber optic channel)  1: **for**
$k=(k^{\prime }+1)\;to\;K\;$ (see Table 3)  2:   Take the observable $Y_{n}^{k}$
  3:    **if** the model is not available **then**  4:    **if** BER > FEC-threshold **then**  5:    Estimate $P({\hat{X}}_{n}^{k}|{\overset{⌢}{Y}}_{n}^{k})$ using $P({\hat{Y}}_{n}^{k-1}|{\hat{X}}_{n}^{k-1})$
  6:    Estimate ${\bar{X}}_{n}^{k}$ by decision making  7:   **else**  8:     Extract the $P({\hat{Y}}_{n}^{k}|{\hat{X}}_{n}^{k})\;\mathrm{and}\;P({\hat{Y}}_{n}^{k})$
  9:      Calculate the posterior $P({\hat{X}}_{n}^{k}|{\hat{Y}}_{n}^{k})$
  10:   Estimate ${\bar{X}}_{n}^{k}$ by decision making   11:  **end if**
  12:  **else if** model is available **then**  13:  Load model, evidence and posterior from preceptor library  14:   Estimate ${\bar{X}}_{n}^{k}$ by decision making  15:  **end if**  16:   Send *PF*_1_, $P({\hat{Y}}_{n}^{k}|{\overset{⌢}{X}}_{n}^{k})\;\mathrm{and}\;P({\hat{Y}}_{n}^{k})$ to the executive  **Internal reward**  17:  Calculate $BER_{k},\;and\;rw_{j}{}^{k}$ and send it to executive  **Planning**  18:  Localize the set of all close actions to *c_k_*  **Learning**  19:  **Apply**
$c_{k+1}$
**virtually (**$c_{k+1}\in C$**)**  20:  Calculate $P(X_{n}^{\left(k+1\right)}|{\hat{Y}}_{n}^{k})$  21:  Predict ${\bar{X}}_{n}^{\left(k+1\right)}$
  22:  Calculate $BER_{k+1}\;and\;rw_{j+1}^{k+1}$
  23:  **if**
$BER^{\left(k+1\right)}\leq threshold$
  and $rw_{j+1}^{k+1}\geq rw_{j}^{k}$
**then**  24:    apply $c_{k+1}$ to the fiber link  25:    j←j+1
  26:    $k^{\prime }\leftarrow k+1$
  27:  **else**  28:    Turn steady state on (Stay on $c_{k}$)   29:  **end if**  30: **end for**

## 5. The CDS case study for the OFDM-Based Long-Haul Standard Single-Mode Fiber system

**Figure 13:**
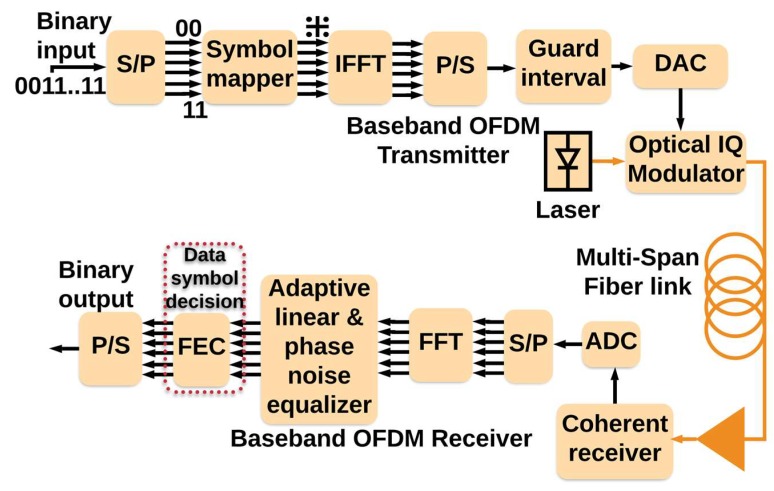
The conventional OFDM-based fiber optic system. (FEC: Forward error correction, P/S: Parallel to serial, DAC: Digital-to-analog converter, ADC: Analog-to-digital converter, S/P: Serial to parallel, IFFT: Inverse fast Fourier transform, FFT: Fast Fourier transform).

**Figure 14:**
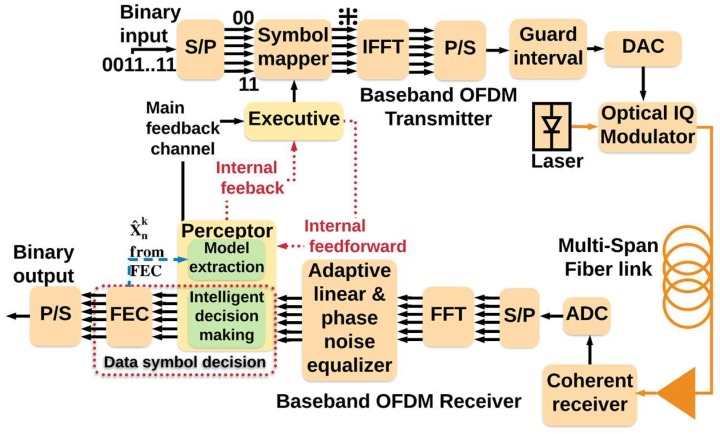
The OFDM-based fiber optic system with simplified CDS.

To illustrate how CDS can control QoS (i.e., increase the data rate/improve the BER), we implemented the CDS with the simplified executive for the OFDM-based long-haul standard single mode fiber link. This is a proof-of-concept case study for the presented Algorithm 1, whose validity to other scenarios will be further studied. The fiber optic system simulation parameters are the same as in Section 3.1.1 (see Table 1), and the parameters of the CDS are as follows: precision factor, PF = 2 and 96% probability box is used. Figure 13 shows the conventional OFDM-based fiber optic system and Figure 14 shows the system with simplified CDS. The detailed describtion OFDM-based fiber optic system are presented in [29,33]. At the transmitter, 256 subcarriers modulated by low data rate QAM-16 data are multiplexed using the inverse fast-Fourier transform (IFFT). Cyclic prefix (CP) is used in the guard interval between OFDM frames to preserve the orthogonality of the subcarriers and also to avoid inter-carrier interference (ICI) due to dispersive effects in the fiber [33]. After digital-to-analog conversion, the OFDM data modulates the laser output using an in-phase quadrature (IQ) modulator. The output of the IQ modulator passes through a fiber optic link consisting of 20 spans of standard single-mode fiber (SSMF) and each fiber span is followed by an inline erbium-doped fiber amplifier (EDFA). The signal propagation in the fiber optic link is governed by the NLSE which includes fiber dispersive effects, loss and third-order nonlinear susceptibility (also known as Kerr effect). The NLSE models the complex envelope of the signal and by solving it, we find the complex envelope of the signal at the output of the link given that the complex envelope of the signal at the transmitter is known. The output of the fiber optic link passes through a coherent receiver in which the OFDM signal is down-converted to the base band. After analog-to-digital conversion, the subcarriers are demultiplexed using the fast Fourier transform (FFT). The adaptive linear equalizer mitigates the linear distortions of the data in each subcarrier in parallel. The phase noise of the transmitter laser and that of local oscillator (LO) are compensated using the standard technique [41]. Now the QAM symbols modulating each subcarrier pass through the perceptor for statistical modeling and intelligent decision-making (see Figure 14). All the figures shown in this manuscript are obtained by simulating the fiber optic system (with or without CDS) using the parameters listed in Table 1.

**Figure 15:**
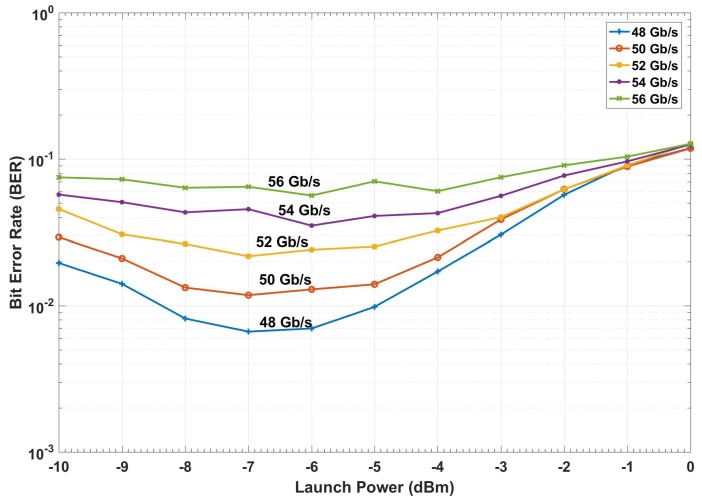
The simulation of a conventional system with a linear equalizer.

**Figure 16:**
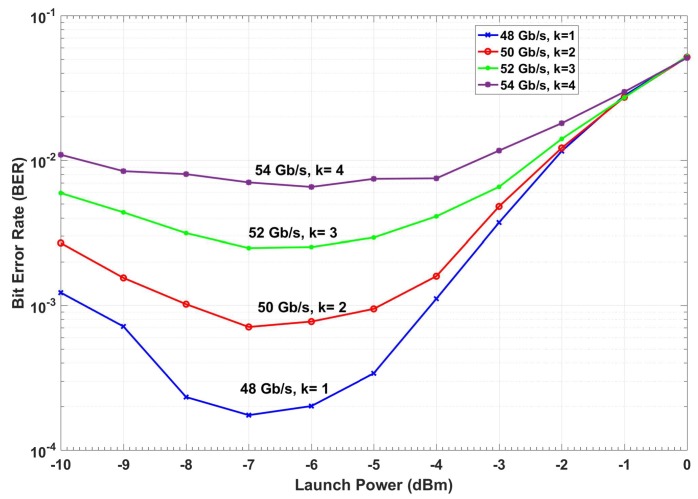
The simulation of a fiber optic system with CDS with the known model (BER improvement mode).

**Figure 17:**
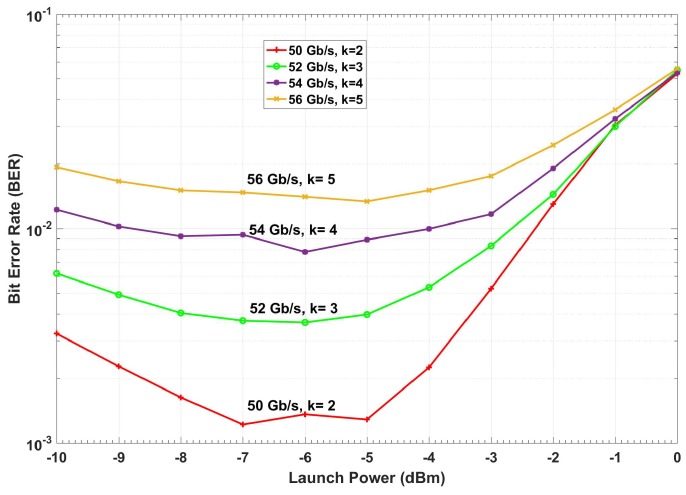
The simulation results for the prospective BER predicted in executive (CDS in prediction mode).

The BER versus launch power of a conventional fiber optic communications system (i.e., without CDS) is shown in Figure 15. The BER is calculated based on the geometric boundaries between the symbols for the conventional system. At low launch powers, the BER decreases with the launch power since the SNR improves. However, at higher launch power (greater than −4 dBm), the BER increases with launch power due to nonlinear impairments. If we consider 1.03 × 10^−2^ (with 14.3% overhead) as the FEC threshold [40], we can see from Figure 15 that the maximum achievable data rate is 48 Gb/s. The adaptive linear equalizer (See Figure 13 and Figure 14) mitigates the linear distortion, and hence, the nonlinear distortion is dominant for data rates greater than 48 Gb/s. The CDS now extracts the model for *k* = 1 (which corresponds to the data rate of 48 Gb/s) in the preceptor while operating in the BER improvement mode (see Figure 16) and saves the model for 48 Gb/s as $P({\hat{Y}}_{n}^{1}\;|{\hat{X}}_{n}^{1})$ in the model library. It may be noted that the BER for the case of 48 Gb/s with CDS (Figure 16, *k* = 1) is lower than the BER of the conventional system at the same data rate (Figure 15) because of the Bayesian approach (see Section 3.1.2). The CDS uses 2 Gb/s discretization step for data rate and a step of 1 dB for the launch power. In the next cycle (*k* = 2), the executive predicts the BER for increasing the data rate to 50 Gb/s using the model $P({\hat{Y}}_{n}^{1}\;|X_{n}^{2})\;$(see Equations (7) and (8)) and is shown in Figure 17. As can be seen in Figure 17, the predicted BER is lower than the FEC threshold and the action of enhancing the data rate is communicated to the transmitter using a low data rate link and transmitter enhances the data rate. Since there is no model in the model library for the case of the data rate of 50 Gb/s, the perceptor will switch to the prediction mode also, and predicts $P({\hat{X}}_{n}^{2}\;|{\hat{Y}}_{n}^{2})$ based on the model for 48 Gb/s (i.e., $P({\hat{Y}}_{n}^{1}\;|{\hat{X}}_{n}^{1})$) and the BER is computed. It is found that the BER is under the FEC threshold as predicted by the executive (See Figure 17, *k* = 2). Since the BER is now below the FEC threshold, the preceptor will operate in BER improvement mode and it will extract the model for *k* = 2 ($P({\hat{Y}}_{n}^{2}\;|{\hat{X}}_{n}^{2})$). Therefore, the BER can be improved further (Figure 16), for example, if we compare the BERs in Figure 16 and Figure 17, when *k* = 2, we find that the BER is lower in the BER improvement mode (See Figure 16) as compared to that in the prediction by the executive (see Figure 17). Since the objective is to enhance the data rate (with BER below the FEC threshold), in the next cycle (*k* = 3), the executive will predict the BER for the enhanced data rate of 52 Gb/s and the predicted BER is shown in Figure 17 (*k* = 3) and this process continues. This process of cycling through the BER improvement mode and prediction mode will continue and the executive will increase the data rate up to 56 Gb/s (*k* = 5 in Figure 17). At this point, the executive finds that the predictive model cannot bring the BER under the FEC threshold (see Figure 17). Therefore, it will decrease the data rate and the steady-state data rate will be 54 Gb/s. The system will continue to operate at 54 Gb/s until there is a disruption to the system. When the disturbance arises, the BER increases and the executive will decrease the data rate, and the perceptor will operate in the prediction mode and choose the closest model from the model library to predict the transmitted data. 

For example, if the gain of an inline amplifier of a fiber optic link drops from 18 to 10 dB, the BER would increase. The BER is sent from the preceptor to the executive that takes action to lower the data rate. The CDS cycles through the prediction mode and BER improvement mode (see Figure 16 and Figure 17) and finally settles down at a data rate that is achievable for the current state of the channel without interruption of the service. The CDS monitors the BER continuously, and if the amplifier is repaired, then the CDS automatically will retrieve the original data rate. Additionally, if there is a cut in a fiber-optic network, the data will be re-routed over a fiber optic link that may have different characteristics such as fiber length, dispersion, and nonlinear coefficient. The CDS senses this change due to the change in BER and again adapts itself to the new environment.

Now, we show the improvement of the BER for the OFDM fiber optic link upgraded with the CDS. It is customary to project the Q-factor based on BER and Gaussian noise statistics as [33,42,43]:

(9) $$Q=20log10\left(\sqrt{2}erfcinv\left(2.BER\right)\right)$$

**Figure 18:**
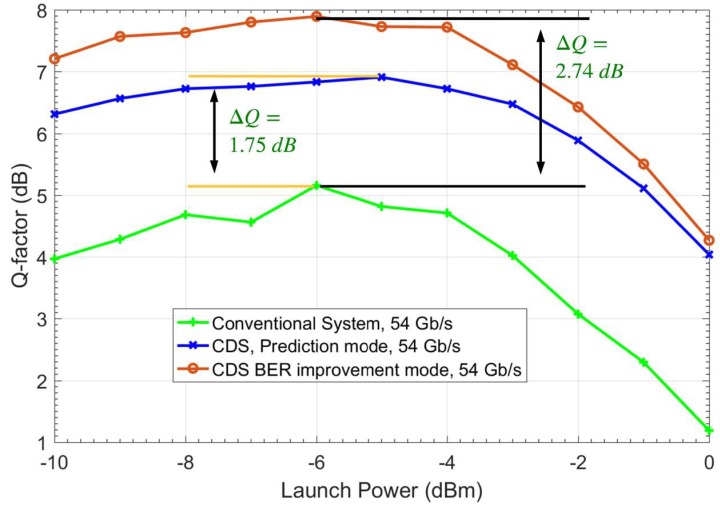
The simulation results for the quality-factor versus launch power for the conventional system, CDS prediction mode, and CDS improvement mode.

In Figure 18, the Q-factors of the conventional system, CDS in BER improvement mode, and CDS in prediction mode for the data rate of 54 Gb/s (*k* = 4) as the function of the launch power are compared. As can be seen, the Q-factor improves by 1.75 and 2.74 dB when the CDS operates in prediction mode and BER improvement mode, respectively. The improvement in BER using the CDS principle is due to the Bayesian approach, while the data rate enhancement is due to the cycling through the prediction mode and BER improvement mode. 

From Figure 18, it may be noted that the optimum launch power does not change for the case of CDS in BER improvement mode as compared to the conventional system, although it increases slightly in the prediction mode (blue curve). However, if DBP is used in a conventional system, then the optimum launch power increases. This is because the DBP compensates for nonlinear impairments to some extent. The simplified CDS in this example should not be considered as a substitute for a nonlinear compensation scheme such as DBP. The difference between the DBP or any nonlinear compensation scheme and our approach is that the DBP makes use of the deterministic channel model (such as nonlinear Schrodinger equation) whereas our approach utilizes the statistical channel model. The Q-factor gain shown in Figure 18 using the simplified CDS is due to the information extracted by the perceptor that helps the CDS to approximate the optimal decision-making boundaries even if the symbol likelihood is non-Gaussian and asymmetric. Therefore, the final data rate is fixed at 54 Gb/s. Additionally, for distances less than 1600 km, we note that the CDS allows sending a signal without coding [44] (soft decision overhead), since the Q-factor is close to the forward-error correction limit (6.75% overhead for hard decision) [40]. Therefore, this reduces the FEC computational cost if the system uses the adaptive FEC and improves data rate efficiency. This Q factor improvement makes the system less sensitive to any small disturbance, and the system will have better QoS with less interruptions.

Using the proposed CDS, the BER can be improved further by operating the preceptor in the BER improvement mode with a lower PF (see Figure 11). However, this is not implemented in the example described here. We have provided an example of how a CDS with a simple executive CDS can still improve the BER, increase the data rate and handle changes in fiber optic channel with less interruption. A complex executive with a combination of different types of actions is still under investigation. From Figure 16, Figure 17 and Figure 18, it may be noted that as the signal power increases, the BER gets larger due to fiber nonlinear impairments. Therefore, the advanced executive should take some advanced actions such as probabilistic shaping or geometric shaping to mitigate the nonlinearity. For example, the CDS can still improve the Q-factor by 3 dB at 0 dBm launch power (Figure 18). However, the Q-factor of 4.2 dB at 0 dBm launch power is below the FEC threshold. Therefore, CDS with advanced actions will be implemented in our future work.

### 5.1. Simulation Results in Presence of Disturbance

**Figure 19:**
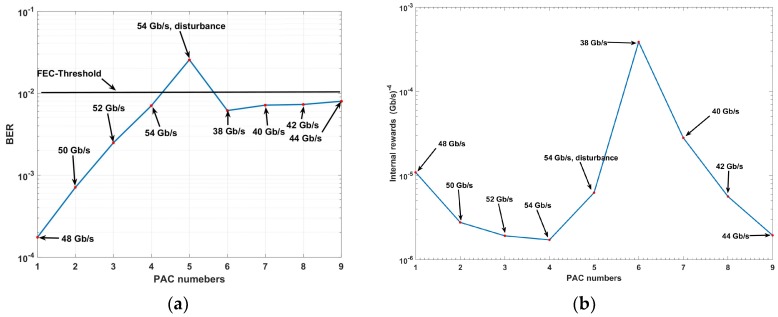
The simulation results for the cognitive dynamic system (CDS) in presence of disturbance (**a**) bit error rate (BER) vs perception action cycle (PAC) numbers, (**b**) CDS learning curve.

If the fiber optic system is disturbed, then the CDS can sense it and adapt to the new environment. We simulate the CDS for the disturbed system by assuming that the tenth inline amplifier of the fiber optic link is partially damaged. The noise figure (NF) of this amplifier is increased from 4.77 to 23 dB, while the rest of the inline amplifiers have the normal NF of 4.77 dB. As a result, the received signal becomes noisier. The simulation results using the CDS in the presence of disturbance are shown in Figure 19. 

Figure 19a shows that in the PAC numbers 1 to 4, the BER is under the FEC-threshold (see Figure 15, Figure 16, Figure 17 and Figure 18), and the CDS achieves a data rate of 54 Gb/s at fourth PAC. Hence, the steady state data rate is 54 Gb/s, and the CDS operates in s steady state mode. At the fifth PAC, the system is disturbed because of the partial damage to the tenth amplifier. The current statistical model for the system operating at 54 Gb/s cannot bring the BER under FEC-threshold. As a result, the CDS turns the steady state mode off and activates the pre-adaptive actions (see Figure 7). Therefore, the CDS lowers the data rate with a step of 2 Gb/s until the BER is under the FEC-threshold. At *k* = 6, *j* is reset to 1, data rate is 38 Gb/s, and the BER is under the FEC-threshold (see Figure 19a). Since $R_{1}^{6}$ = 38 Gb/s, $R_{ref}$ is updated to $R_{1}^{6}$ − *d* = 36 Gb/s. The procedure for PAC numbers 6 to 9 is similar to that for PAC numbers 1 to 4 (see Algorithm 1). Additionally, the rewards are calculated using Equation (4), and Figure 19b shows the internal reward $rw_{j}^{k}$ as a function of the PAC number *k* calculated. Before disturbance (i.e., *k* < 5) we see that the internal reward decreases as *k* increases. This is because the weight of the data-rate is more than the weight of the BER (see Equation (4)). The executive would increase the data rate if the internal reward is decreasing (provided that the BER is less than the FEC-threshold). At *k* = 5, there is a disturbance leading to an increase in the BER and hence, internal reward also increases. The internal reward reaches its peak at *k* = 6 since the difference between $R_{1}^{6}$ and $R_{ref}$ is the lowest (see Equation (4)). Thereafter, the internal reward decreases at *k* = 7, the CDS applies the same procedure used at *k* = 2. At ninth PAC number (*k* = 9), the new steady-state data rate in the presence of disturbance is the 44 Gb/s (similar to fourth PAC number). If the amplifier is repaired in future, the CDS follows the same procedure as in PAC numbers 1 to 4 to increase the data rate again.

### 5.2. CDS Complexity

In order to calculate the computational cost, we distinguish three types of CDS modes: (i) BER improvement mode, (ii) prediction mode, and (iii) steady state. The complexity of a CDS varies depending on the perceptor mode. When the CDS operates in the BER improvement mode, the BER converges after *N* OFDM frames (in Figure 12, it is about *N =* 512) and each frame has *K* data-carrying subcarriers (From Table 1, it is 126). To create the histogram (i.e., to calculate $P(Y_{n}^{k}|X_{n}^{k})$) based on the discretized data, we need *N* × *K* simple additions (just counting the samples within the tiny surface). To calculate $P(X_{n}^{k}|Y_{n}^{k})$, we need *M* real multiplications where *M* is the order of QAM (in our example, *M* = 16). The storage requirement depends on the precision factor (see Table 2). For example, with a PF of 2, we need to store a matrix of dimension 81 × 71 × 16. In addition, we need to store the matrix corresponding to $P(X_{n}^{k}|Y_{n}^{k})$ which has a dimension of 16 × 81 × 71. When the CDS operates in the prediction mode, there is no cost associated with model extraction (i.e., to calculate $P(Y_{n}^{k}|X_{n}^{k})$), but we need *M* real multiplications to calculate $P(X_{n}^{k}|Y_{n}^{k})$. In this mode, we need to store the matrix corresponding to $P(X_{n}^{k}|Y_{n}^{k})$, which has a dimension of 16 × 81 × 71. In steady state, the CDS uses the saved $P(X_{n}^{k}|Y_{n}^{k})$, and makes a decision symbol by symbol (see Equation (3)). These matrices can be stored in hard disk and can be loaded to RAM when needed. No multiplication or addition needs to be done in steady state. 

The computational cost associated with DBP is as follows. In each propagation step, $J\log_{2}(J)$ complex multiplications are required where *J* is the number of samples in time domain. If there are *B* propagation steps, the complex multiplication is $O(B\times J\log_{2}J\;)$ [20,21]. For example, for a 20-span system, assuming 10 steps per span and with *J* = 16,384, we need roughly 45,875,200 complex multiplications! Besides, this computation needs to be done continuously for each block of data. Three complex vectors of length *J* (about the total size of 98,304) needs to be stored. In contrast, most of the computational complexity of the CDS is present only when there is a fluctuation in the fiber optic channel; under steady state, the computational cost is minimal. It is useful to compare the complexity of the proposed method in this paper with the digital backpropagation (DBP) for Q-factor enhancement using the simulation parameters shown in Table 1. We run both algorithms on a Microsoft Surface-Pro with Intel^®^ Core™ i706650U CPU @ 2.20GHz 2.21 GHz, 16 GB RAM, system type 64-bit Operating System x64-Based processor using MATLAB. For the DBP algorithm [21], the runtime is 1225.4 s (~20 min and 25 s). However, the runtime for the proposed CDS is 14.03 s for PF *= 2*. In addition, the CDS runtime reduces to 7.71 s for PF *= 2* in steady state mode. However, the DBP running time always remain similar for any continuous data stream. In addition, the reported Q-factor improvement for DBP in OFDM systems is between 1 and 2.2 dB [45,46], while it is 2.74 dB (see Figure 18) for the fiber optic link upgraded with the CDS. Additionally, the main features of the proposed CDS are the following:

1. The CDS perceptor can extract the model (likelihood) dynamically and does not need the transmitter to send training sequences.
2. We assume that the fiber optic link parameters are unknown in an optical network.
3. The CDS can be applied to any type of fiber optic system such as single carrier, wavelength division multiplexing (WDM), single channel OFDM, or a WDM system with individual channels carrying the OFDM signal.
4. In our approach, we do not use training sequences from the transmitter to extract the channel model $P(Y_{n}|X_{n})$ since it would disrupt the service. It can improve the BER by finding the optimum decision boundaries.

## 6. Conclusions

The cognitive dynamic system (CDS) is inspired by the human brain and can be regarded as the brain-like intelligence. The principles of CDS are applied to a nonlinear fiber optic communication system for the bit error rate (BER) improvement and the data rate enhancement. The executive and preceptor of CDS are implemented in the optical receiver, but executive sends actions to the transmitter such as new data rate. The block diagram of the CDS consisting of the preceptor, the main feedback channel, and executive is presented. The preceptor operates in two modes: prediction mode and BER improvement mode. When there is a fluctuation in the fiber optic network parameters, such as reach or amplifier gain, the BER may exceed the forward error correction (FEC) threshold. In this case, the preceptor operates in prediction mode and, working closely with the executive, it lowers the BER to meet FEC threshold. Once the BER comes below the FEC threshold, the perceptor operates in the BER improvement mode. In this mode, it extracts a statistical model for the fiber optic channel for the current fiber optic link parameters and saves it in the model library for future use. By cycling through the prediction mode and BER improvement mode with appropriate actions by the executive, we have found that the data rate and Q-factor can be enhanced by 12.5% and 2.74 dB, respectively, as compared to the conventional fiber optic system. The key advantage of CDS is that it is intelligent, and software-defined, and it can automatically recognize and extract information about the fiber optic channel. In addition, it can enhance the data rate and improve the BER for different fiber optic link characteristics such as the span length, input power, and other system/signal parameters. These characteristics may change during the data transmission in a fiber optic network, but the CDS can still adapt to these changes. The computational cost of the proposed technique is much lower than the methods used to mitigate nonlinear impairments, such as digital back propagation (DBP). It should be noted that the simplified CDS implemented in this paper is not a substitute for nonlinear compensation techniques such as DBP, although it provides performance benefits comparable to that obtained by DBP. The future application of CDS for fiber optic communications system will include advanced actions such as probabilistic and geometric modulation shaping to mitigate nonlinear distortion in conjunction with the current perceptor, which is expected to improve the BER further and enhance the maximum achievable data rate.

## Figures and Tables

**Table 1:** The numerical simulation parameters of the orthogonal frequency-division multiplexing (OFDM) system.

Simulation Parameters	Value
Fiber dispersion coefficient ($\beta_{2})$	$-22.1{\;\mathrm{ps}}^{2}/\mathrm{km}$
Fiber nonlinear coefficient (γ)	$1.1{\;W}^{-1}{\mathrm{km}}^{-1}$
Fiber loss coefficient (α)	0.2 dB/km
Number of fiber spans (*N*)	20
Span length (*L_span_*)	80 km
Noise figure (NF)	4.77 dB
Number of OFDM subcarriers per frame	256
Subcarrier modulation	QAM-16
Guard intervals (cyclic prefix)	1.86 ns
Number of data frames	1024
Line width of transmitter laser/LO	22 kHz
Data carrying subcarriers per frame	126
Over sampling factor	2
Number of pilot subcarrier	2
Wavelength (λ)	1550 nm
Length of PRBS	$2^{16}-1$
Fiber type	Standard single mode fiber (SSMF)

**Table 2:** $P({\hat{Y}}_{n}^{k}\;|{\hat{X}}_{n}^{k})$ system model dimension versus precision factor (PF) extracted by simulation (number of OFDM frames = 1024).

Precision Factor (PF)	$P({\hat{Y}}_{n}^{k}\;|{\hat{X}}_{n}^{k})$ System Model Tensor Dimensions
5	33×29×16
2	81×71×16
1	161×141×16
0.5	321×281×16

**Table 3:** The important notation list used in this paper.

Notations	Value
*K*	The total number of environmental perception action cycles (PACs)
*k*	The current PAC number during PAC running
*k*′	The PAC number before the current CDS steady state mode
*C*	The set of all possible actions in actions library
X	The set of all possible modulation state
$X_{n}^{k}$	The current transmitted symbols, at time *n*
${\overset{⌢}{X}}_{n}^{k}$	The known data using for modeling
${\bar{X}}_{n}^{k}$	The estimated decision by the system
$Y_{n}^{k}$	The received symbols at time *n* and *k*th PAC
${\bar{Y}}_{n}^{k}$	The normalized received symbols at time *n* and *k*th PAC
${\overset{⌢}{Y}}_{n}^{k}$	The discretized received symbols at time *n* and *k*th PAC
$rw_{j}^{k}$	The internal reward at time *n, j*th data rate, and *k*th PAC
$P({\hat{X}}_{n}^{k}|{\hat{Y}}_{n}^{k})$	The estimated posterior at time *n* and *k*th PAC
$c_{k}$	Current action applied on fiber optic link
FEC−threshold	The predefined threshold
$P(X_{n}^{\left(k+1\right)}|{\hat{Y}}_{n}^{k})$	The prediction using model at *k*th PAC
$rw_{j+1}^{k+1}$	The internal reward prediction
$c_{k+1}$	The final action for applying in (*k*+1)th PAC
